# Health care providers’ perspectives of diet-related non-communicable disease in South Africa

**DOI:** 10.1186/s12889-020-8364-y

**Published:** 2020-02-21

**Authors:** Jo Hunter-Adams, Jane Battersby

**Affiliations:** 1 Health Economics Unit, School of Public Health and Family Medicine, University of Cape Town, Anzio Road, Observatory, Cape Town, 7925 South Africa; 2 African Centre for Cities, University of Cape Town, Rondebosch, Cape Town, 7700 South Africa

**Keywords:** Diet-related non-communicable disease, South Africa, Health care providers

## Abstract

**Background:**

In South Africa, diet-related non-communicable diseases (dr-NCDs) place a significant burden on individuals, households and the health system. In this article, we investigate the experiences of eight key informants within the public sector health care system (nurse, doctor and dietician), in order to reflect on their experiences treating dr-NCDs.

**Methods:**

We interviewed eight key informants who were central to the primary care service for at least 40,000 people living in a low-income neighbourhood of Cape Town, South Africa. In previous work, we had interviewed and conducted ethnographic research focused on dr-NCDs in the same neighbourhood. We then conducted a thematic analysis of these interviews.

**Results:**

The perspectives of key informants within the public sector therefore offered insights into tensions and commonalities between individual, neighbourhood and health systems perspectives. In particular, the rising prevalence of dr-NCDs alarmed providers. They identified changing diet as an important factor driving diabetes and high blood pressure in particular. Health care practitioners focused primarily on patients’ individual responsibility to eat a healthy diet and adhere to treatment. A marked lack of connection between health and social services at the local level, and a shortage of dieticians, meant that doctors provided rapid, often anecdotal dietary advice. The single dietician for the district was ill-equipped to connect dr-NCDs with the upstream determinants of health. While providers often had empathy and understanding of patients’ circumstances, their training and context had not equipped them to translate that understanding into a clinical context. Providers seemingly could not reconcile their empathy with their perception of dr-NCDs as a failure of prudence or responsibility by patients. Significant shortcomings within health systems and social services make reflexive practice very difficult.

**Conclusions:**

Supporting health care providers in understanding context, through approaches such as translational competency, while strengthening both health and social services, are vital given the high burden of NCDs in South Africa.

## Background

Diet-related non-communicable diseases (dr-NCDs) burdens the South African public health system, as part of a quadruple burden of disease [1]. Dr-NCDs affect quality of life and entail a significant cost to health systems, the economy and households. Abegunde et al. [2] conservatively estimate the accumulated losses to South African gross domestic product (GDP) because of diabetes, stroke, and coronary heart disease at US$1.88 billion, for the period between 2006 to 2015 [3].

Despite their considerable impact, the public sector is not sufficiently responsive to many NCDs. For example, a recent study found that over 89% of diabetes patients in a South African in a nationally representative sample had significant unmet medical needs [4]. NCDs are often preventable and therefore framed as diseases of lifestyle. However, there is little evidence of successful behavioural interventions to change lifestyles. The limitations of individually focused interventions suggests that public health policymakers must translate the social determinants of health and disease [5], outlined in a food context by the UNICEF [6], into practice. Health care providers, policymakers and researchers must reduce these frameworks into tangible steps to improve care and reduce health inequity.

Our previous research focused on the food and NCD experiences of residents within a low-income neighbourhood. In this work, we found that participants had complex relationships with weight [7], which were at odds with dominant discourse relates obesity to lack of prudence, or to lack of knowledge. Dr-NCDs seemed to relate in important ways with a lack of dietary diversity, and with food insecurity [7, 8]. Given how this research suggested that individual behavioural interventions would do not address the lived experience of dr-NCDs amongst patients who lived in this neighbourhood, we wanted to gather the perspectives of health care providers’ who treated these patients. The goal of this paper is therefore to understand the scope and limitations of health systems responses to dr-NCDs in one local context. While the South African food system, health system, and economic context are unique, we will also reflect on ways in which these contexts may resonate for international readers. We situate this article within broader questions of how health care providers might navigate individual behavioural change considering the significant social and systemic drivers of NCDs. This relates to issues of cultural [9], structural [10] and translational competency [11] within the health care system, which we attempt to draw out in one, local context.

## Methods

### Background and context

We base the results on an analysis of semi-structured interviews with 8 key informant health care providers affiliated with three facilities closest to the low-income neighbourhood sampled, focusing on one clinic and one hospital that treated the NCDs of most residents. The interviews focused on health care providers’ perspectives of diet-related non-communicable diseases (dr-NCDs) in a low-income urban neighbourhood of South Africa.

We selected key informants based on having relevant experience treating dr-NCDs amongst residents of this neighbourhood. JHA conducted all interviews in English. The key informants included five medical doctors, two nurses, and one dietician. While the patient population is primarily black, seven of the eight key informants were white (Table 1). JHA interviewed the current doctor at one facility, and the nurse practitioner, and the recently retired doctor from the other clinic. The dietician was the only dietician practicing in the public sector in this health district and saw patients at multiple facilities, including one hospital (inpatient and outpatient) and four clinics. In addition, JHA interviewed three doctors and one nurse practitioner in the hospital setting. Each doctor played a somewhat different role in the hospital.

**Table 1:** Key informant interviews

Facility	Key informant identifier in results	Key informant role	Key informant race	Key informant gender
Hospital	Doctor 1	Primary care clinic doctor (Medical officer)	White	Male
	Nurse 1	Primary care walk-in nurse manager	White	Female
	Dietician	Dietician	White	female
	Doctor 2	Primary care clinic doctor (registrar)	White	Male
	Doctor 3	Casualty doctor	Mixed race	Male
Clinic 1	Doctor 4	Medical doctor	White	Female
Clinic 2	Doctor 5	Medical doctor	White	Female
	Nurse 2	Nurse practitioner	Black	Female

We designed the interview guide to be responsive to the time constraints of health care practitioners in the public sector. Doctors at these primary health care clinics typically treated forty patients in just 4 h. The interview guide (as presented in Appendix) began with questions focused on experiences of patients with NCDs, including HIV and TB co-infection, questions about patients’ diets, and assessment of the supports available to patients and to health care providers to support patients with, or at risk for dr-NCDs. The research questions that we were interested in answering focused on perceptions of patient knowledge of diet and NCDs, perspectives on support structures or lack thereof for patients with NCDs, and recommendations for improved care.

JHA transcribed interviews verbatim. We read all transcripts multiple times for overall meaning and key issues emphasized by participants. We then conducted a thematic analysis [12], and coded interview transcripts manually using Hyperresearch (Researchware Inc., 2009, MA, USA). The themes mapped to interview questions and were deductive.

Approval from the Research Committee of the Provincial Government of the Western Cape, the City of Cape Town, and the Human Research Ethics Committee, Faculty of Health Sciences, University of Cape Town (HREC 098/2016). Written informed consent was obtained from all participants.

## Results

In conducting our thematic analysis, we outlined six themes related to diet and NCDs for healthcare providers (1) Widespread acknowledgement of diet-related NCDs as a serious concern (2) Perception of patients’ lack of knowledge of diet-related NCDs (3) Perspectives on the limited role of the health care provider (4) Perceptions of lack of safety nets (5) Perceptions of the role and constraints of the health care system. (6) Recommended responses to non-communicable disease.

### Widespread acknowledgement of diet-related NCDs as a serious concern

Health care providers were very concerned about a perceived increase in the prevalence of non-communicable disease and expressed sentiments about obesity and NCDs concurrently. Providers expressed concern over co-morbidities, particularly high blood pressure and diabetes:

*Every patient just about has some kind of co-infection. If they’re diabetic obviously they’re going to have ischemic heart disease.* Doctor 2

*I can just see it [NCDs]! The young people coming in 30-year olds with heart attacks, 28 year olds Type II diabetic. Twenty year old type II diabetic but “oh but he’s young!” but he’s fat! and that’s the problem, so that if you start your life at 20 with Type 2 diabetes well then within 9 years 10 years you have end stage disease already. Because it takes 9 years to really manifest fully.* Doctor 3

*like Type II Diabetes is the new scurge it’s gonna be gonna cost us millions and millions and millions and it’s totally preventable!* Doctor 3

Concern over obesity tended to be expressed as a proxy for concern over the burden on NCDs:

*I see very .... as much as there’s people that have no food there are people that are grossly overweight. I mean I have patients that weight 160 kg that come to the clinic* Doctor 5

*The majority... A lot of our HIV patients are far too obese, and they are there, they are obese because they WANT to be obese, because they don’t want to be seen as thin and suffering from HIV. Now, it is very difficult to tell, even a diabetic, that “You are obese.” Why? Because people are defensive.* Doctor 1

The strong sentiment was that obesity was a marker of bad dietary choices, and therefore NCDs, were in some way related to defaulting on personal responsibility. In particular, the second quote articulates the providers belief that individuals “want” to be obese in order to assert that they are HIV-negative.

The expression of concern around dr-NCDs was palpable, as well as providers’ sense that poor diets were causing NCDs, and that these were linked to poor knowledge and poor choices, as expanded upon in the next section.

### Perspectives on patients’ lack of knowledge of diet-related ill-health: prudence and restraint

Providers generally felt that patients had little to no knowledge of diet and NCDs:

*When you’re dealing with more first-world kind of people you assume they know these kind of things, but [neighbourhood name] people don’t know it. um. I mean a lot of diabetic patients have not had that counselling. so sometimes it just helps to teach them what the difference is between a coke zero and coke... little things like that.* Doctor 5.

Where patients had poor diets that resulted in obesity and non-communicable disease, they considered this in terms of lack of self-control.

*I said that it’s mind over matter.* Doctor 1

*Now I’m talking about obese patients and that there might be something else that’s triggering it, because at the end of the day, it does become a mind thing of telling yourself “should I have these chips or should I rather have these vegetables or whatever the case may be.”* Dietician

However, the practitioners did also consistently acknowledge financial constraints.

*And the problem is we don’t have the dietary guidelines... the dietary guidelines don’t match up to what the people can actually afford. There’s a disjoint because we haven’t really cottoned on that you know we have to target diets to particular groups of people who can afford or who cannot afford it so it’s no good telling people salmon is great… You can’t afford that.* Doctor 3

*Also, so also when I try to counsel them about they should and shouldn’t ... should eat more and shouldn’t eat... also the financial implications of that.... they can’t afford to.... eat.... I mean it’s easy to make yourself a gigantic bowl of rice and just have a little bit of sauce with it. It’s a much cheaper way to eat... than having 3 different kinds of vegetables… and some chicken. So I think a lot of the time the things that they eat are what they can afford...also they don’t always know, what is good and what is not um. but. I think also .... if you only have R5 are you going to buy a head of lettuce or are you going to buy an amagwenya (*Fried dough, sometimes with filling)*?* Doctor 2

While acknowledging the financial challenges of a healthy diet, health care providers felt that a nutritious diet could be affordable, in that they emphasized the expense of soda/soft drinks relative to more nutritious (but lower calorie) foods, re-emphasising issues of self-control.

*I find a lot of them would rather go buy a 2 L coke, with their money, even though they don’t have much, and drink that, where you can say, instead of using that, go to... because even in [neighbourhood] I’m sure you’ve seen it they have those little stores with fruits and vegetables that are much cheaper compared to in the shops, so even just telling them how to work with that kind of money, and make those little changes as well.* Dietician

*I show my love for my family if I can treat my whole family to a meal at KFC, which is just poisonous chicken. ok. It is so awful for them, and they all love it. And that to them, eating what they call luxuries is a sign of a wealth and a sign of... of... um... I’ve made it in the world. Whereas we’re all trying to get back to the basics where the Eastern Cape actually kept them healthy in the first place... we want to try and get back to that.* Nurse 1

While medical doctors seldom provided written dietary recommendations, nursing staff and the single dietician interviewed both provided various fliers with simple dietary information. This information was simple and easy to read and recommended that individuals consume specific foods in greater quantities and avoid others. However, it is notable that handouts were tailored towards a specific goal, weight loss, or towards a specific complaint, such as reflux, OR high blood pressure, OR diabetes. However, the health care providers understood that most people had a complex mixture of needs, goals, and immediate concerns, which could not be engaged in the brief clinical encounter or simple dietary information.

### Perspectives on the limited scope of provider role around diet

Only one of the health care providers had a clear and direct role in relation to diet, but even then, only for patients with specific needs. Core to the dietician’s job was explaining the illness and recommending diets for diabetic patients. Given the scarcity of resources and time, she could only focus on those who seemed able and willing to make dietary changes:

*I see where they are at, and if they are willing to make these changes, and if they are even willing to make these changes, so then unfortunately because we’re so limited with resources, I can’t waste my time with someone who is not interested.* Dietician

The dietician would attempt to adjust diets based on her understanding of possible financial adaptations that might be appropriate:

*Obviously if they don’t have a lot of things I try to adapt by saying ok with what you have now, try to do this or make these changes, and try to just do the best... it is difficult... but try to do the best that I can with what they have, but again it’s always looking at social backgrounds and seeing if they need to be referred to social workers.* Dietician.

The other interviewed health care providers saw their roles in advising patients on their diets as very circumscribed:

*So, as a clinician, right, my job, is NOT to talk about diet, not to talk about diet, unless I felt I need to reinforce that a patient needed to follow a diet.* Doctor 1.

This constrained role seemed to be shaped by the limited time and limited perceived impact of provider-patient interactions:

*It’s so depressing, it’s so depressing, it’s so depressing because you really want to change people, change people’s view of the world and you can’t in the 10, 15 min that you spend with them. So a lot of the time you do your whole talk and you think you’re connecting and you say “so can you tell me what I’ve just been talking about? Like blank. OK... So we got nowhere, and I just give up.* Doctor 3

The role of providers were profoundly informed by the limitations of training, capacity, and by health systems challenges.

### Lack of safety nets

Health care providers were acutely aware of their patients’ needs, and acknowledged the widespread dietary needs in the community. They noted an absence of NGO and state safety nets to support patients.

*I don’t have too many places to send them, when I find something… We have a lot more population in our area and whereas before the food fund fed maybe xyz monthly, now it’s not xyz anymore, it’s xyz and their entire families.... um... so that food just doesn’t.... and the funding seems to have dried up...* Nurse 1

*So [NGO name] provides Food parcels. And then the um.... what do you call it. and the place used to give the already cooked food.... they’ve apparently stopped that? …But it’s sad because those were the only two options we knew of. So we have a lot of people who just DON’T have anything to eat* Doctor 5*.*

Referral to social services was a viewed as a problematic safety net. Providers repeatedly raised the limited scope and very limited responsiveness of Department of Social Development as a concern.

*the people don’t have a lot of faith in Social development, in the social workers...not at all. I have absolutely zero faith in the social workers. Because I’ve referred patients there .... several times.... I never get feedback. They NEVER phone me back. I have to drive there and physically go and talk to them if I want anything done...* Doctor 5

This perception of lack of state or other safety nets meant that referrals were unlikely to yield meaningful support to patients. In this context, providers expressed insights relating to the role of the health system, and the health care providers.

### Perspectives of health systems’ constraints

Health care providers described several health systems issues as central to patients’ experiences of NCDs, namely: continued prioritisation of infectious diseases; short appointment times, complex health needs, provider turnover, and language barriers.

One provider noted.

*it’s for us as clinic it’s a much higher priority for them to go and recall the patients that are failing their ARVs or not coming to their appointments, rather than having them do BP checks. [sure] so in our community... probably our priority... like our priority is still with communicable diseases... with TB and HIV... and the priority hasn’t quite shifted to there yet.* Doctor 5

The capacity of the single dietician was extremely limited. Not only was just one, newly qualified dietician responsible for all patients in a hospital and three clinics, but until this year the post was a community service post, which meant that the facilities had had a different newly qualified dietician each year. The dietician therefore had a large caseload and significant responsibility with very limited support: This meant that one new dietician had a large caseload and very significant responsibility:

*Especially coming into straight after university and there not being another dietician here, and no-one really handing over to you... so you’re just like how am I going to do this? Where do I start?! so it did take a few months and then I eventually got a system going…*Dietician*.*

The interviewees working in the hospital setting noted that continuity of care between practitioners was important for the treatment of NCDs, but was not possible given structural constraints:

*The problem that we have is... and we’re addressing it... is that up until recently ... the patient never saw the same doctor. … what we’re trying very hard to do [now] is to have patients see the same doctor each time so there is a consistent pattern as to how that patient is.* Doctor 1

The challenges of continuity of care was also between hospital and clinic care. Municipal government typically administer primary care clinics, whereas provincial government administers larger hospitals. Health care providers described challenges related to attempting to create continuity of care between the two structures. Providers found it complicated to address referral needs and making sure pharmacy filled prescriptions correctly. For example, one doctor reported the need for diabetic referrals to optometry, while another referred to prescriptions being cancelled at one pharmacy, yet continued at another:

*maybe someone’s on treatment at their local clinic and then we discover “oh! this person actual has renal failure let’s change your meds [….]so then there’s a disconnect because now we have stopped these medicines but then they go to the clinic and they keep on collecting them […] then they came in again with a second hypoglycaemic episode which is potentially dangerous... life threatening…* Doctor 4

NCD patients who were stable could also receive medication in NCD adherence clubs, which were run in a government-non-governmental partnership:

*like for the clubs they come twice or three times for the blood visit or the clinical visit when we do a physical check-up and then we take bloods once or two times a year, and then for the other visits they just go and collect their medication. Sometimes they don’t even come to the facility. so it’s good they go to the* [NGO name] *and just collect their medication.* Nurse 2.

Providers often found it difficult to discuss illness, diet or treatment in any depth with patients, because of language barriers, given that most providers received just 1 year of language training in isiXhosa and Afrikaans.

*… the population here at [hospital] is quite diverse … we have Somalis and Malawians and Mozambicans and Zimbabweans.... um.... and um. We see this mix of people along with the Xhosas and coloured people and the white people so we have a lot of diversity.* Doctor 2

### Provider recommendations

When asked for recommendations to improve NCDs, providers described a range of potential interventions, ranging from dietary education in the clinic waiting room, to urban agriculture, to BMI taxation. More than being necessarily practical interventions, these recommendations reveal that health care providers’ view of dr-NCDs involves prudence, choice, and lack of motivation: where they feel patients needed to be educated to make better dietary choices, that poor individuals should pursue urban agriculture, and even that high BMI is enough of a choice to warrant taxing those with higher BMI.

While initially discussing subsidization of healthy food, another provider shifted to suggesting the value of taxing obesity, which again assumes a high level of choice related to obesity:

*Japan has a weight tax... so you pay extra tax depending on your BMI, depending on your waist circumference ...I wonder how that will go down in this country. Rather than taxing things like petrol ....* Doctor 2

This recommendation has class implications given that wealthy individuals have access to highly palatable healthy food, and opportunities to exercise. Providers expressed a sense that choice drove good diets and healthy weights when they emphasized the need for patient education. Providers made concrete suggestions for affordable, brief educational interventions in the mornings at one clinic:

*I think the education is a big thing… I mean little things. I mean if someone with more knowledge could do a little talk in the mornings... because we need .... when you get to* [neighbourhood] *at 8 o’clock, there’s a hundred people sitting. So I mean it would be such a little thing for a dietician or someone to come and do a general talk about healthy eating, and just to replace... small things ... white bread with brown bread... just little things like that.... um. I mean I think that would make a big difference.* Doctor 5

Similarly, providers suggested gardening, and keeping hens, while acknowledging space constraints. This recommendation on the one hand highlighted the potential of eggs as protein, but also downplayed the role of the built environment and living environment in shaping and constraining the consumption of fresh produce.

*I think it would be wonderful if... if ... more people could grow their own stuff... and it’s proven that in a postage stamp type garden you can actually be .... if you plant carefully... you can actually be quite good. um... it would be wonderful if people keep their own two hens. To lay, eggs. Um. because I really don’t think meat is that important as a ...I think the veggies is far more important... and rather going for more grain type things... and beans. Bean type protein.* Nurse 1

It was evident that some health care providers were grappling with significant tensions in this perspective. For example, when discussing wellness and exercise programmes, a provider simultaneously raised the issues of safety:

*I think there are ... there are many potential things... I know at some point the national health was trying do like a wellness thing and at various hospitals staff and patients started doing some exercise and that sort of thing. so you could have you could have if you could train get the physio to run a small class and train people how to run a class you could run at every church and every community hall some sort of exercise programme, on a regular basis. Because some people have the impression that going to the gym is the only way to get exercise. But it also depends what community you’re in because in [neighbourhood name], people fear for their lives if they walk in the streets I have had people tell me quite openly I know I’m supposed to exercise but I don’t and I can’t ... because people get shot. If you walk in the street you get shot. and it’s like ... it’s not far from the truth.* Doctor 2

Only once did a provider discuss structural change, and the need for significant structural support for patients with very low incomes, and this was only when asked direct follow up about social development:

*Oh yes competent social development people. Really I feel the commitment is not there. Honestly... most of the nurses will not refer a patient... to social worker... they’ll come and ask me because they don’t even know who they are, where they are, what the number is. and I mean that’s terrible. Because I mean in a clinic that’s where we would...that’s where all the problems go [and then um in terms of uh there being opportunities for soup kitchens and food parcels, the food parcels are very haphazard right now] and very much appreciated when they are available* Doctor 5

This recommendation highlighted the need for very basic safety nets in the community, including basic food parcels. Nevertheless, overall health care providers’ recommendations suggest underlying perceptions of obesity or poor diet as a choice, or patients being generally unaware of their poor choices.

## Discussion

We recognize the limitations of the specific local context in which we conducted our research, and the small number of key informants who form part of the core NCD providers in the neighbourhood. The findings described by health care providers suggest a dissonance between the experience and perceptions of community members and health care provider responses to dr-NCDs. Our results reflect the challenge of bridging the gaps between the prevailing framing of individual behaviour change for dr-NCDs on the one hand, and clear systemic causes of dr-NCDs, on the other. Providers reproduced medicalized narratives of exercising prudence and restraint and extending knowledge related to diet. Popular culture reinforced these ideas. Providers’ daily work experiences yielded unique and deep insights into of the health impacts of poor living conditions in the neighbourhood, yet providers could not process or act on this knowledge. We therefore want to discuss this tension in which providers are trained to treat dr-NCDs as diseases of lifestyle, while they were deeply cognizant of broader environmental context that shape patients’ behaviour. We will suggest that translational competence [11] as one framework for bridging this divide, and discuss whether there are ways to “translate” translational competence for providers.

In previous research amongst residents of the neighbourhood, there was an understanding of the urgency of dr-NCDs within the neighbourhood. Residents described their pressing needs in relation to uncertainty around food, housing, and work, and felt these impacted on their impressions and experiences of weight. Experiences of weight were complex, nuanced, and involved much care around the body [7]. The health of the body was a core area of competency, and an area where individuals focused attention and interest [7, 8]. Health was directly linked to income, where most residents did not receive pay on sick days or needed a doctors’ certificate to access sick leave, which involved long clinic wait times and potentially unnecessary visits. Participants expressed strong motivation to pursue good health and engage in preventive dietary practices, given the loss of income involved with dr-NCDs. In particular, patients highlighted the many ways in which dr-NCDs were related to the food system, to transportation, to employment or lack thereof. In contrast, health care providers framed dr-NCDs as a problem related to lack of knowledge and poor choices. We highlight potential opportunities to bridge this gap, in the next section.

### Bridging the gap between individual behaviour change and the social determinants of health

First, health provider perspectives focused on framing dr-NCDs as a problem of individual knowledge, prudence and responsibility by patients, even as they acknowledged collective dietary changes at the neighbourhood and national level. Providers were acutely aware of, and alarmed by, the pressing social and environmental needs in the neighbourhood their patients lived in, including experiences of hunger. Our concern is that health care providers did not have the tools to connect their perspective of dr-NCDs to the broader socio-economic, and environmental context they witnessed first-hand in this low-income neighbourhoods.

While health practitioners could identify some structural barriers to the effective engagement with dr-NCDs, their articulation of the problem and its potential solutions showed entrenched ways of thinking about illness and patients. In the South African context issues of race, class and cultural difference play a vital role in the experience of the health system by both patients and providers. While South African medical training includes language classes and community service modules, we argue—aligned with Yates-Doerr’s [11] framing—that the medical system needs to extend beyond attention to cultural competency, which is the focus of current South African medical training. We suggest that cultural competency, structural competency, and translational competency represent progressively more complex frameworks for empathy and support by health care providers.

Cultural competence has been described as involved five parts: of cultural awareness, cultural knowledge, cultural skill, cultural encounters and cultural desire [13]. In some ways, the health care providers in this study reflected a model of care focused on perceived cultural norms, including related to weight. Yet Anthropologist Arthur Kleinman and colleagues have problematized this approach, because of the ways it may imply that an individual is defined by their race, nationality or ethnicity, or imply that providers may successfully treat patients by following a set of checkboxes [9]. In a South African context cultural competency is problematic, because it sets up patients and providers in terms of difference.

Although our understanding of the training at South African medical schools does not include all institutions, we know that Western Cape-based medical schools require instruction in local languages, and in the social determinants of health. Motivational interviewing attempts to remove paternalism from a health care provider’s approaches, because it frames health behavioural change in terms of patients’ own willingness to change. This is necessarily reductionist, because it shifts responsibility onto the patient but the patient must still act to prioritize health over other, potentially important, concerns.

There is a need to extend beyond cultural competence towards structural and translational competence.

Structural competence adherents argue that providers need to understand structural inequalities into clinical medicine, and explicitly understand the role of the social and economic determinants of health, and their own blind spots and biases (Metzl and Hansen, [10]). The goal of structural competence would be to foster empathy and humility amongst health care providers.

Ultimately, however, we argue that health providers need to move towards translational competence, described by Yates-Doerr as “joining concern for cultural differences with concern for structural inequity, and understanding how medical structures, which are tied to histories of violence, colonialization, and dispossession, will value some cultures over others” (Yates-Doerr, [11], p266). This seems to offer a route through which to move away from the moral weight that is associated with patient non-compliance in relation to NCDs. However, determining how to teach and practice translational competence is difficult, as it implies that knowledge, rather than context, makes it difficult for health care providers to practice empathy. Our research suggests that health care providers felt deeply for their patients, yet the stressful health systems context, and lack of social safety nets, made the tenets of translational competency very difficult to translate into practice.

The development of these competencies among practicing and training practitioners is necessary, but not sufficient to address the health system’s engagement with patients with dr-NCDs. The failure of social services and the overcrowded health system impacts directly on patient care, and providers’ powerlessness given structural problems seemed to reinforce reductionist thinking. Health systems policy makers and those engaged in medical teaching should couple an understanding of translational competence with the prioritization of health systems connections to other public sectors. Individual health care providers form part of broader structures.

The single dietician working in this area had very limited capacity to apply their training to low-resource contexts, or to serve the very large patient population within her jurisdiction. Given the high prevalence of dr-NCDs nutritional knowledge should be a core competency for a broader range of health care providers, and cannot be the sole domain of dieticians. As the burden of NCDs in South Africa increases, clear dietary and nutritional support need to be an integral part of medical training, and/or dietician positions need to be far more plentiful in the public sector. The current vacuum in nutrition training results in nurses and doctors drawing heavily on their own nutrition beliefs, which are often anecdotal and not evidence based. Doctors’ empathy for their patients is in tension with comments that suggest strong anti-fat bias.

## Conclusions

Medicalized interventions focused on individual dietary behaviour change do not address the lived realities of low-income patients. Where providers perceive diet to be a matter of choice, they arrive to a clinical encounter with negative perceptions of their patient, as they perceive the patient to have chosen their illness. Approaches that focus on “mind over matter” do not engage the significant structural and social determinants of who gets sick, how they experience their illness, and how their families cope with their family members’ chronic illness. These questions, and their answers, are key questions asked by health care providers. It is vital that health systems help providers grapple with these structural and social determinants of health to reduce paternalism and improve patient care.

## Data Availability

Participants did not give consent for full transcripts to be made available to the public.

## Appendix

### Health care provider interview question guide

1. What is your position at the clinic?
2. How long have you had this position? (prompt: What position did you have before this)
3. Where did you receive your medical training?
4. When did you receive your medical training?
5. Can you tell me a little bit about your experiences treating patients with non-communicable disease? (follow up prompts: experiences with HIV co-infection? TB co-infection? During pregnancy?)
6. Could you please talk a bit more about your patient’s eating habits, to the extent of your experience?
  - If they feel people should choose differently, ask about hunger, success/lack of success choosing.
  - What kinds of supports are there for patients here?
7. Could you talk a bit about your experience talking to patients about non-communicable disease? (follow up prompts: What kinds of advice do you think has been most helpful to patients? What do you think has been least helpful? Have you received feedback from patients?)
8. How do you think the clinic could support patients more?
9. What kinds of help/support/services might be helpful outside of the clinic?
10. Is there anything else you’d like to share about food and non-communicable disease in the patients you see?

## Abbreviations

dr-NCDs: diet-related non-communicable disease
NCDs: Non-communicable disease

## References

[1] Mayosi BM, Flisher AJ, Lalloo UG, Sitas F, Tollman SM, Bradshaw D (2009). The burden of non-communicable diseases in South Africa. Lancet.

[2] Abegunde DO, Mathers CD, Adam T, Ortegon M, Strong K (2007). The burden and costs of chronic diseases in low-income and middle-income countries. Lancet.

[3] Hofman K (2014). Guest editorial: non-communicable diseases in South Africa: a challenge to economic development. S Afr Med J.

[4] Stokes A, Berry KM, Mchiza Z, Parker W, Labadarios D, Chola L (2017). Prevalence and unmet need for diabetes care across the care continuum in a national sample of south African adults: evidence from the SANHANES-1, 2011-2012. PLoS One.

[5] WHO Commission on Social Determinants of Health, World Health Organization (2008). Closing the gap in a generation: health equity through action on the social determinants of health: Commission on Social Determinants of Health final report.

[6] United Nations Children’s Fund (2015). UNICEF’S approach to scaling up nutrition: discussion paper.

[7] Hunter-Adams J (2019). Perceptions of weight in relation to health, hunger, and belonging among women in periurban South Africa. Health Care Women Int.

[8] Hunter-Adams J, Battersby J, Oni T (2019). Food insecurity in relation to obesity in peri-urban Cape Town, South Africa: implications for diet-related non-communicable disease. Appetite..

[9] Kleinman A, Benson P (2006). Anthropology in the Clinic: The Problem of Cultural Competency and How to Fix It. PLoS Med.

[10] Metzl JM, Hansen H (2014). Structural competency: theorizing a new medical engagement with stigma and inequality. Soc Sci Med.

[11] Yates-Doerr E. Translational competency: on the role of culture in obesity and interventions. Med Anthropol Theory. 2018;5:106-17.

[12] Braun V, Clarke V (2006). Using thematic analysis in psychology. Qual Res Psychol.

[13] Campinha-Bacote J (2002). The process of cultural competence in the delivery of healthcare services: a model of care. J Transcult Nurs.

